# Supplementary figures and images for: Metabolome and Transcriptome Unveil the Correlated Metabolites and Transcripts with 2-acetyl-1-pyrroline in Fragrant Rice
Source: Int J Mol Sci. 2024 Jul 27;25(15):8207. doi: 10.3390/ijms25158207 (PMC11311731; doi:10.3390/ijms25158207)

**A**

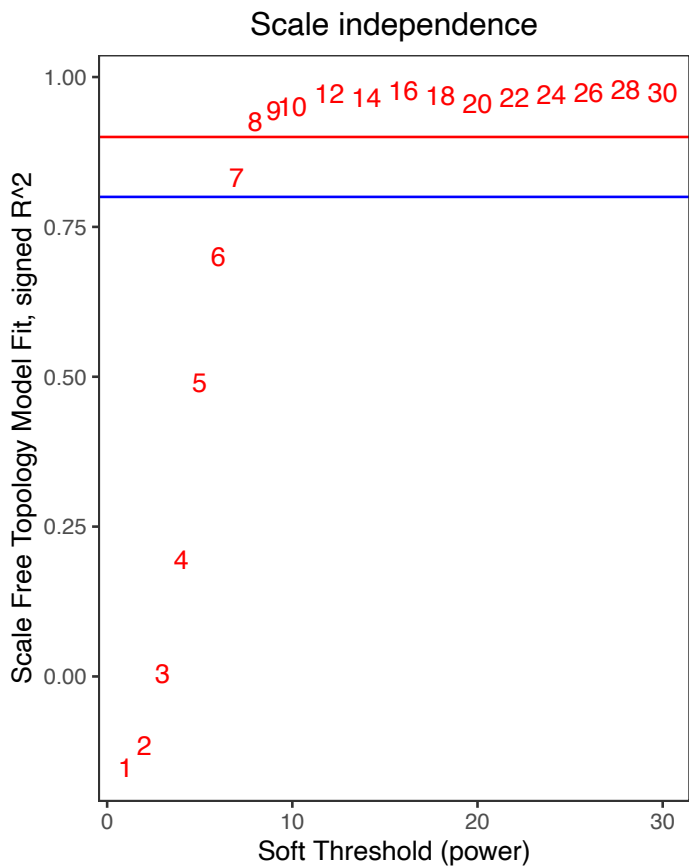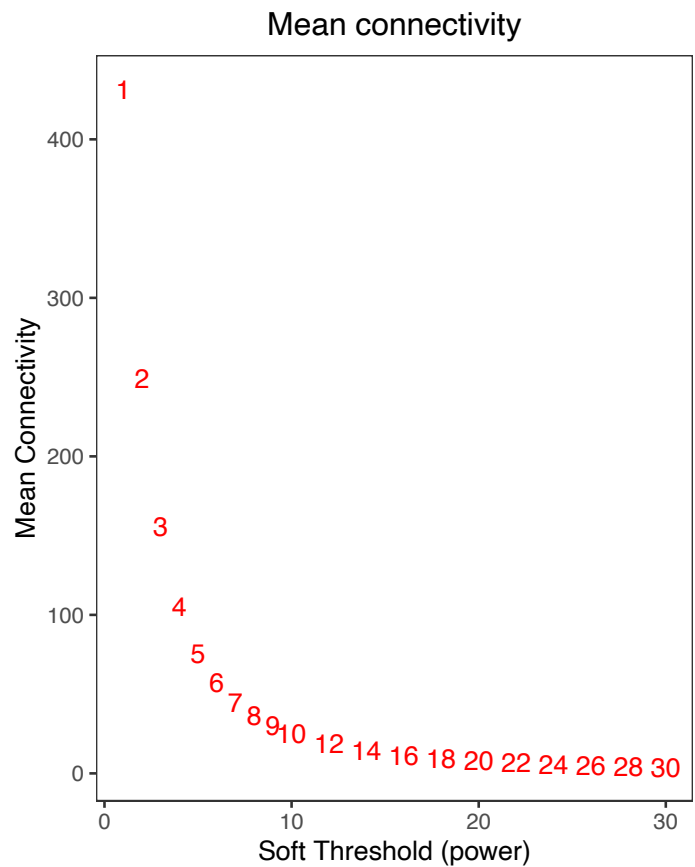

**B**

### Cluster Dendrogram

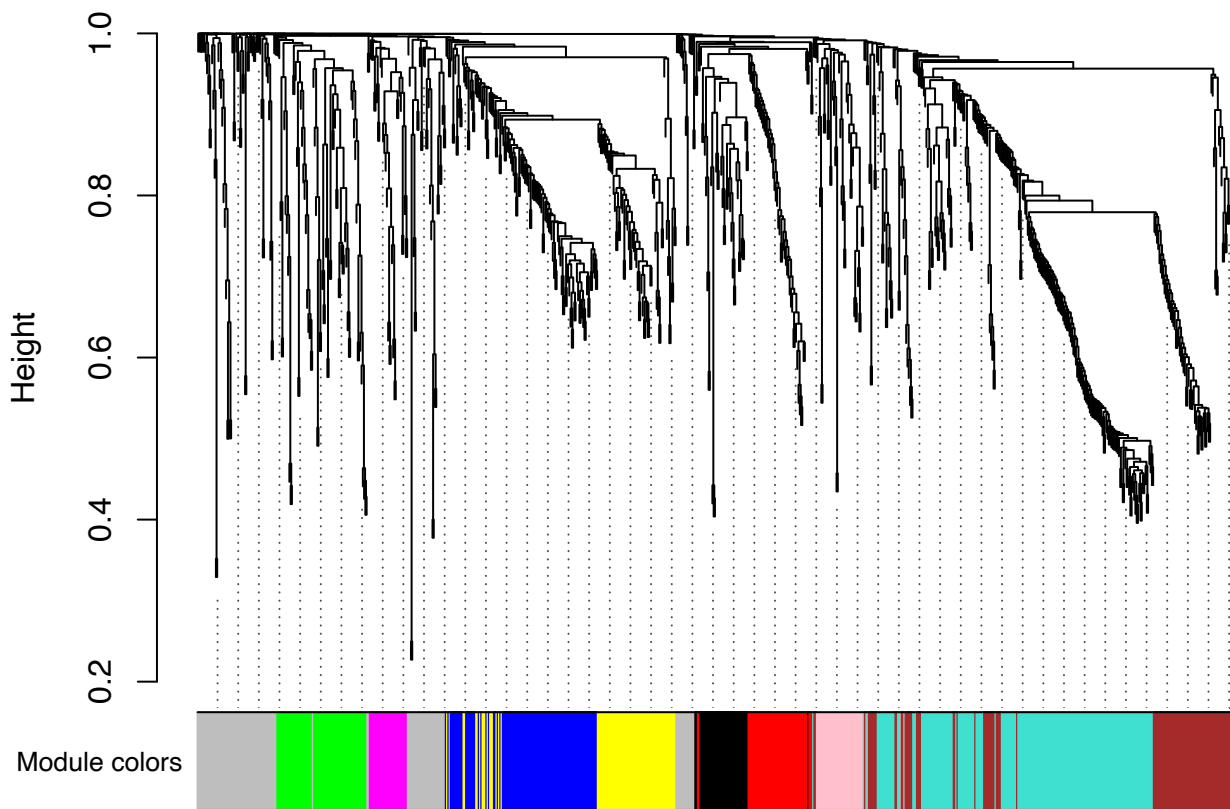

Supplement: Supplementary file 1 [file ijms-25-08207-s001.zip › New Suppl Figure S1.pdf]

**A****Scale independence**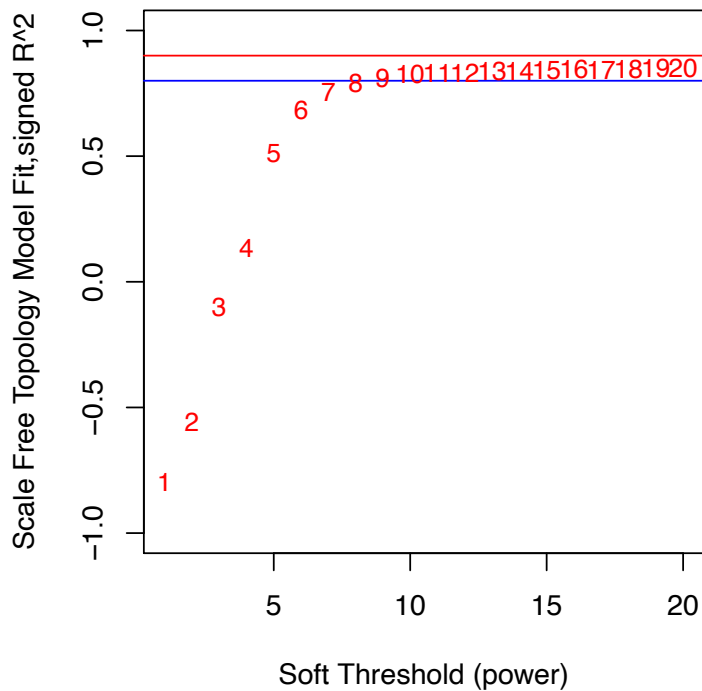**Mean connectivity**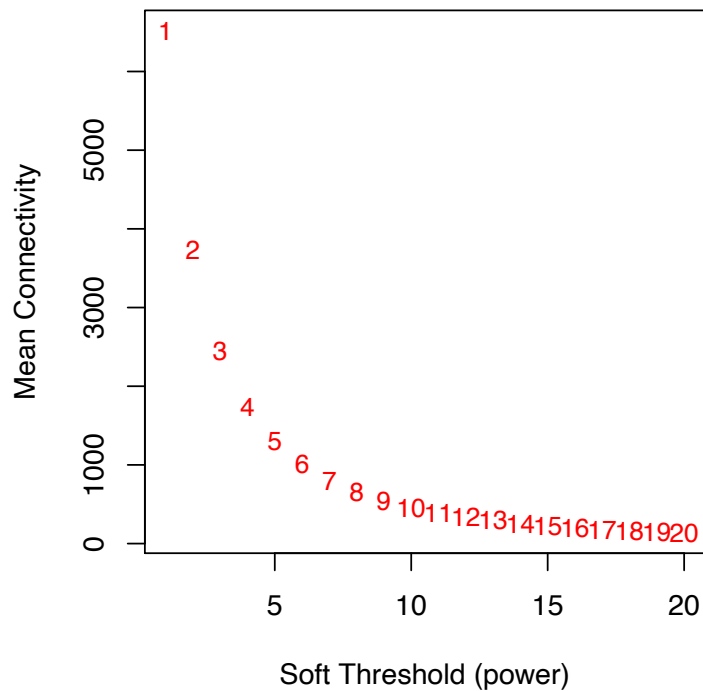**B****Cluster Dendrogram**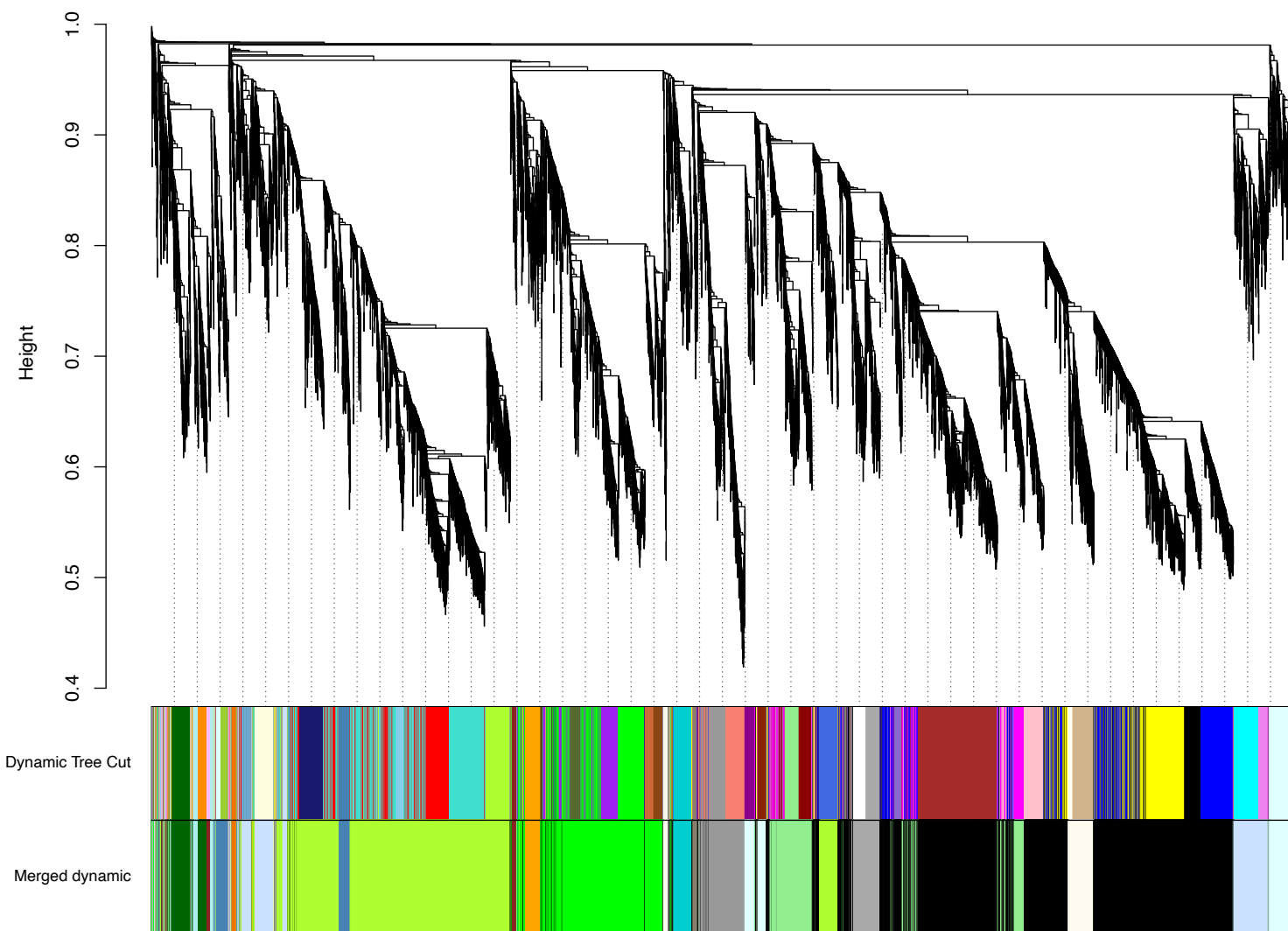

Supplement: Supplementary file 1 [file ijms-25-08207-s001.zip › New Suppl Figure S2.pdf]

**A**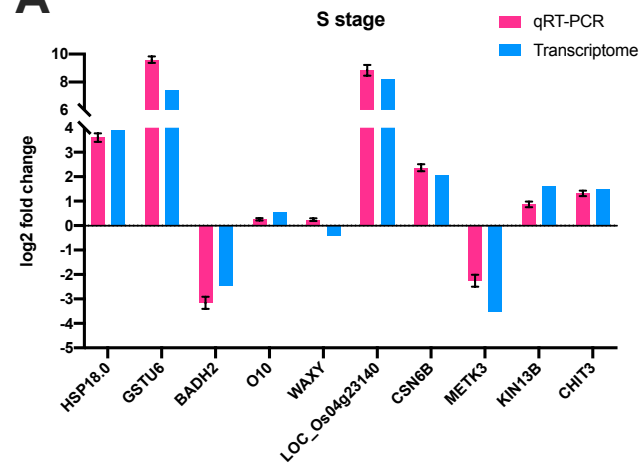**B**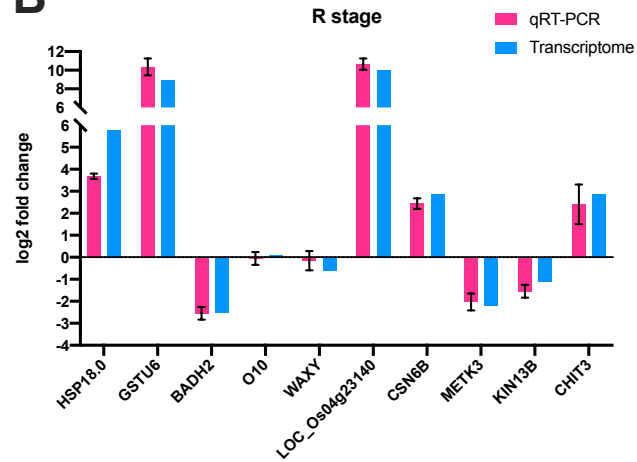**C**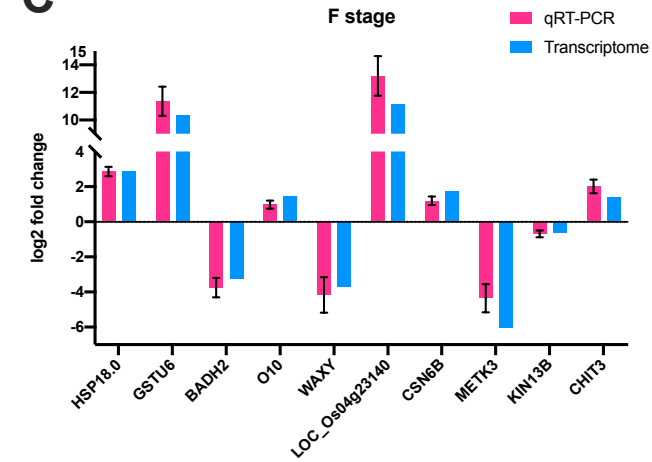**D**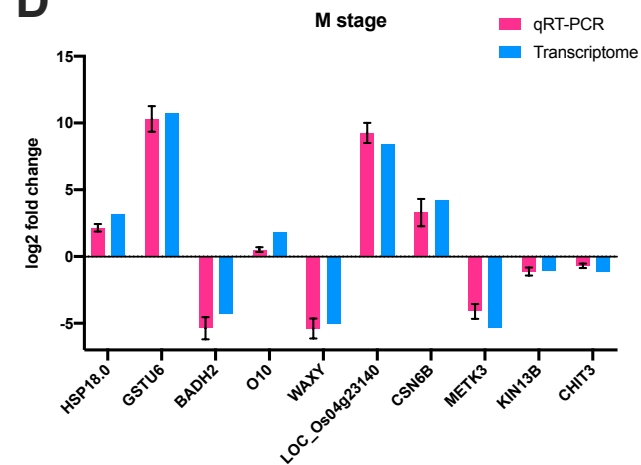**E**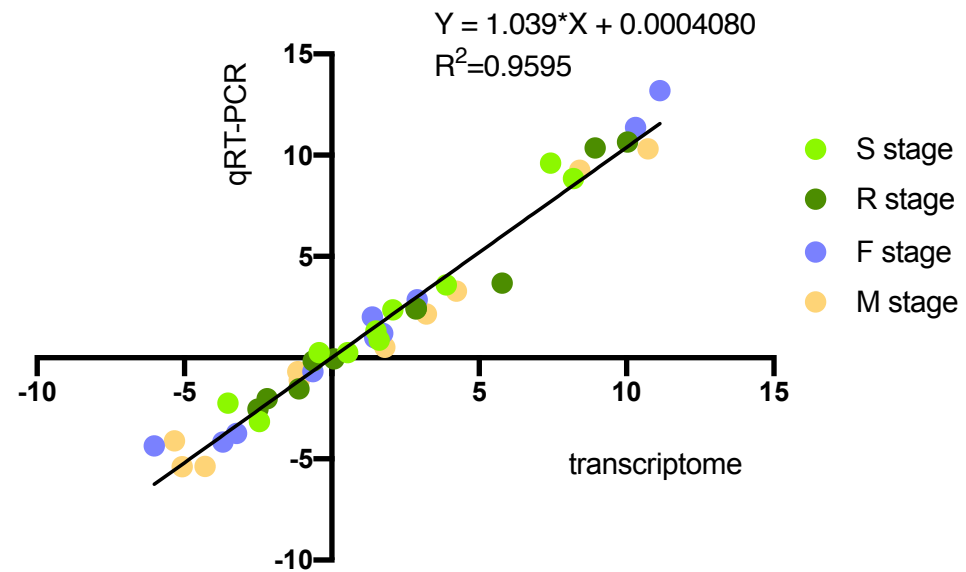

Validation of Transcriptome using qRT-PCR

Supplement: Supplementary file 1 [file ijms-25-08207-s001.zip › New Suppl Figure S3.pdf]
